# Detecting industrial motor faults with current signatures

**DOI:** 10.12688/f1000research.54266.1

**Published:** 2021-09-08

**Authors:** Shashikumar Krishnan, Vijayakumar Vengadasalam

**Affiliations:** 1 Faculty of Engineering, Multimedia University, Cyberjaya, Selangor, 61000, Malaysia; 2 Faculty of Computing and Informatics, Multimedia University, Cyberjaya, Selangor, 61000, Malaysia

**Keywords:** Hilbert Transform, AI, 3-phase, Induction Motor

## Abstract

**Background:** A major player in industry is the induction motor. The constant motion and mechanical nature of motors causes much wear and tear, creating a need for frequent maintenance such as changing contact brushes. Unmannered and infrequent monitoring of motors, as is common in the industry, can lead to overexertion and cause major faults. If a motor fault is detected earlier through the use of automated fault monitoring, it could prevent minor faults from developing into major faults, reducing the cost and down-time of production due the motor repairs. There are few available methods to detect three-phase motor faults. One method is to analyze average vibration signals values of V, I, pf, P, Q, S, THD and frequency. Others are to analyze instantaneous signal signatures of V and I frequencies, or V and I trajectory plotting a Lissajous curve. These methods need at least three sensors for current and three for voltage for a three-phase motor detection.

**Methods:** Our proposed method of monitoring faults in three-phase industrial motors uses Hilbert Transform (HT) instantaneous current signature curve only, reducing the number of sensors required. Our system detects fault signatures accurately at any voltage or current levels, whether it is delta or star connected motors. This is due to our system design, which incorporates normalized curves of HT in the fault analysis database. We have conducted this experiment in our campus laboratory for two different three-phase motors with four different fault experiments.

**Results: **The results shown in this paper are a comparison of two methods, the V and I Lissajous trajectory curve and our HT instantaneous current signature curve.

**Conclusion: **We have chosen them as our benchmark as their fault results closely resemble our system results, but our system benefits such as universality and a cost reduction in sensors of 50%.

## Introduction

There are many common causes of motor failures that happen in industry, such as voltage imbalance, transient voltage, harmonic distortion, sigma currents, operation overloads, shaft voltage and many more. A three-phase induction motor is the most common, significant machine used in industry compared with direct current (DC) motors. ^1^

This induction motor is exposed to a variety of conditions that will cause motor failure such as shaft imbalance, which will subsequently reduce its lifetime. Therefore, faults such as a fractured rotor bar and irregular air gap eccentricities must be detected at the earliest stage possible ^2^ to prevent the motor from sustaining worse damage and deteriorating in performance. ^3^ There are many conventional methods available in order to maintain a motor's condition; for example, an engineer or technician might carry out scheduled check-ups in order to inspect the condition of a motor and rectify any faults. One of the fault detection systems available in the market is the Motor Current Signature Analysis (MCSA).

The fundamentals of applying MCSA, ^4^ the current and voltage waveform, will be analyzed to produce the power signal known as motor signatures. The obtained unique waveform will help the user in distinguishing and sorting out the healthy from the unhealthy motors. ^5^ MCSA is able to detect various types of faults such as air gap irregularities, which are divided into static, dynamic or both. ^6^ Throughout recent years, the advancement of Appliance Load Monitoring (ALM) has been proposed to carry out particularized energy sensing and to give information on the separation of the energy. ^7^ ALM was classified into non-intrusive ^8^ and intrusive ^9^^,^^10^ load monitoring. Normal electrical and mechanical conditions which lead to machine failure need to be tracked. ^11^ In previous years, Fast Fourier Transform (FFT) of MCSA was widely used in detecting faults. Then, a new method called Park’s vector method was proposed for further investigation of motor faults. ^12^ Some methods involving artificial intelligence had been used for identifying faults by using fully automated pattern recognition. ^13^^,^^14^ In literature ^15^ active real power signatures have been used as the basics of fault identification, while other research has used raw single-cycle current, instantaneous power and admittance spectrum for this purpose. ^16^ V-I trajectory is a measurement that refers to voltage and current waveform in implementing nomenclature of device signatures. ^17^

This project was conducted to detect and monitor the faults for three-phase machines, which will help technicians and engineers working in industry in detecting the faults. This would also help in improving the maintenance and productivity of an industry. We designed this project by applying artificial intelligence to distinguish between a healthy and faulty electrical machine in industry, which would help create an easier to use and cost-effective design. A minimum of 10 training data samples of motor faults that have minimum variations from the actual data are used for comparison and analysis. ^18^ Convolution Neural Network (CNN) is used for disaggregation purposes and is often used in computer recognition. ^19^ Hilbert Transform (HT) application in this project has been helpful in detecting faults with clear visualization by using a minimum number of sensors. We designed a three-phase machine monitoring and pre-fault detection system to aid engineers for preventive maintenance. By the end of this project, we were able to monitor and detect the healthy condition of the three-phase machine, and to differentiate each of the faults occurring and propose a suitable artificial intelligence method to solve the faults.

## Methods

### Phase one: data acquisition VT and CT

**Figure 1.**
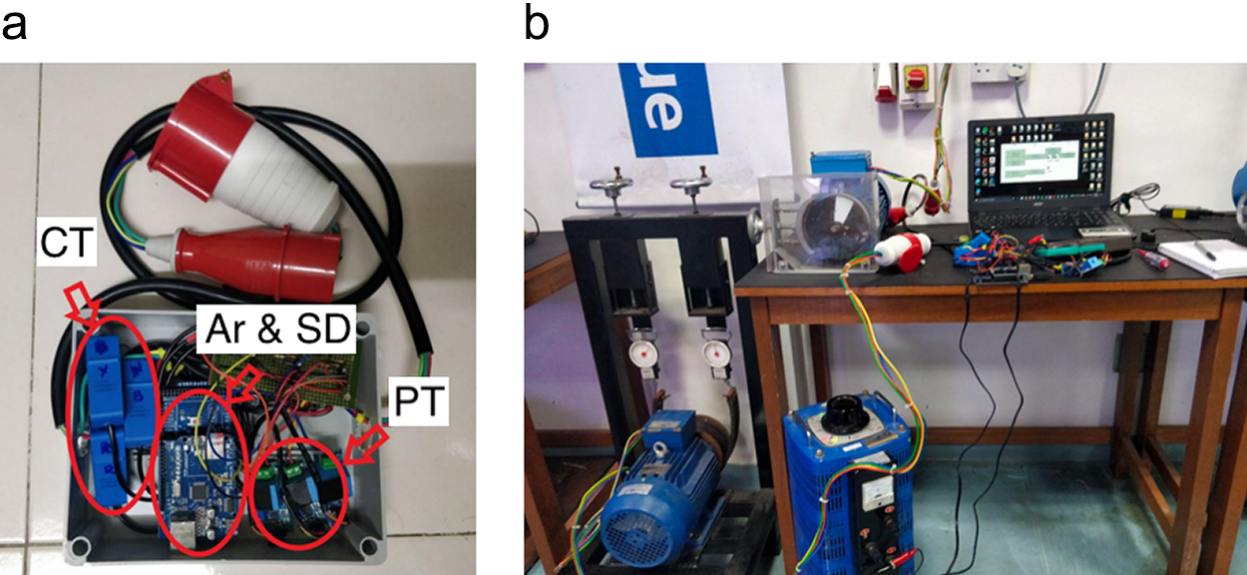
Hardware setup.

There are two types of load monitoring ^7^ non-intrusive load monitoring (NILM) ^8^ and intrusive load monitoring. ^9^ We are using the non-intrusive type as it does not need to physically hijack the wires of the three-phase motor, granting ease of installation with plug and play. The microcontroller we have chosen is Arduino Mega 2560 R3 which is suitable to use as a high-speed logger for the 50Hz power signals. ^20^ The Arduino was inserted with ethernet shield W5100 module and micro SD card, AC potential sensor 2mA single phase transformer ZMPT101B and the NILM split-core AC current sensor SCT 013-030 rated 30A manufactured by YHDC as shown Figure 1. This Arduino Mega hardware setup was combined with implementation of Arduino Software IDE Version 1.8.15 for coding and programming. The high sampling rate of 8500 samples/second per input were taken.

**Figure 2.**
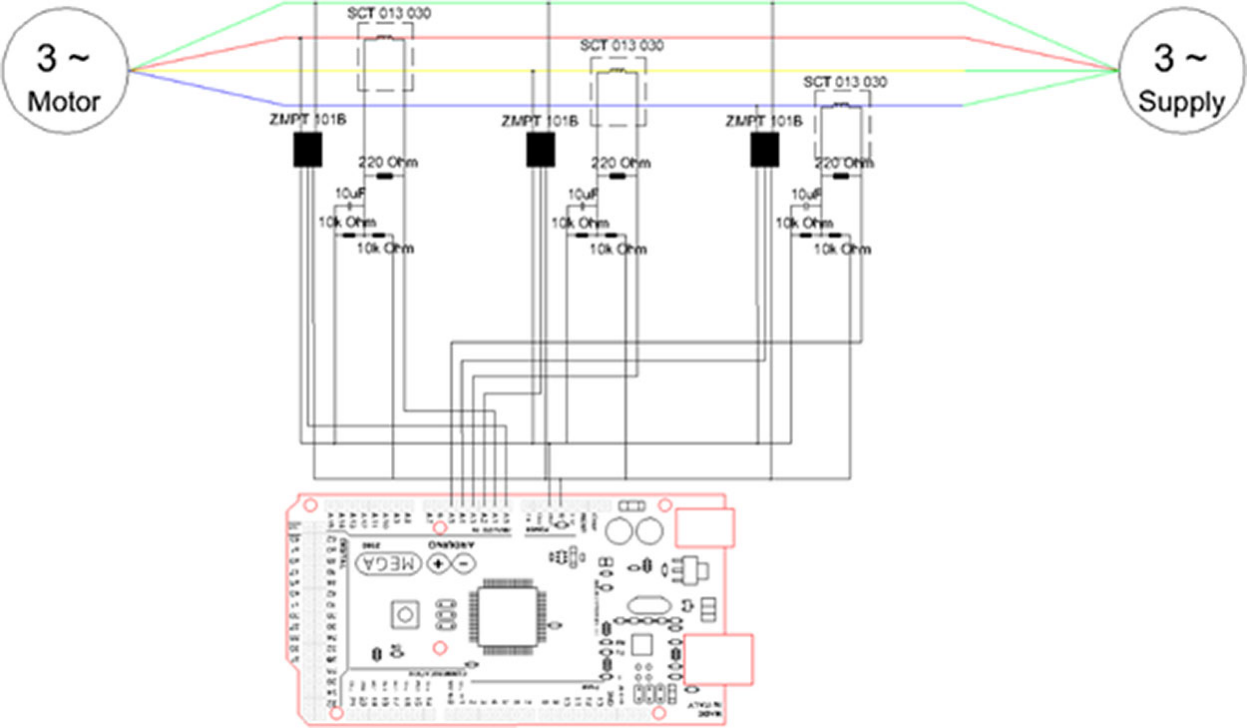
Schematic diagram.

The hardware system used in Figures 1 and 2 is for benchmarking two methods: V and I Lissajous trajectory curve, and our HT instantaneous current signature curve. Our system alone would result in deleting the VT sensors.

### Phase two: appliance features extraction

**Figure 3.**
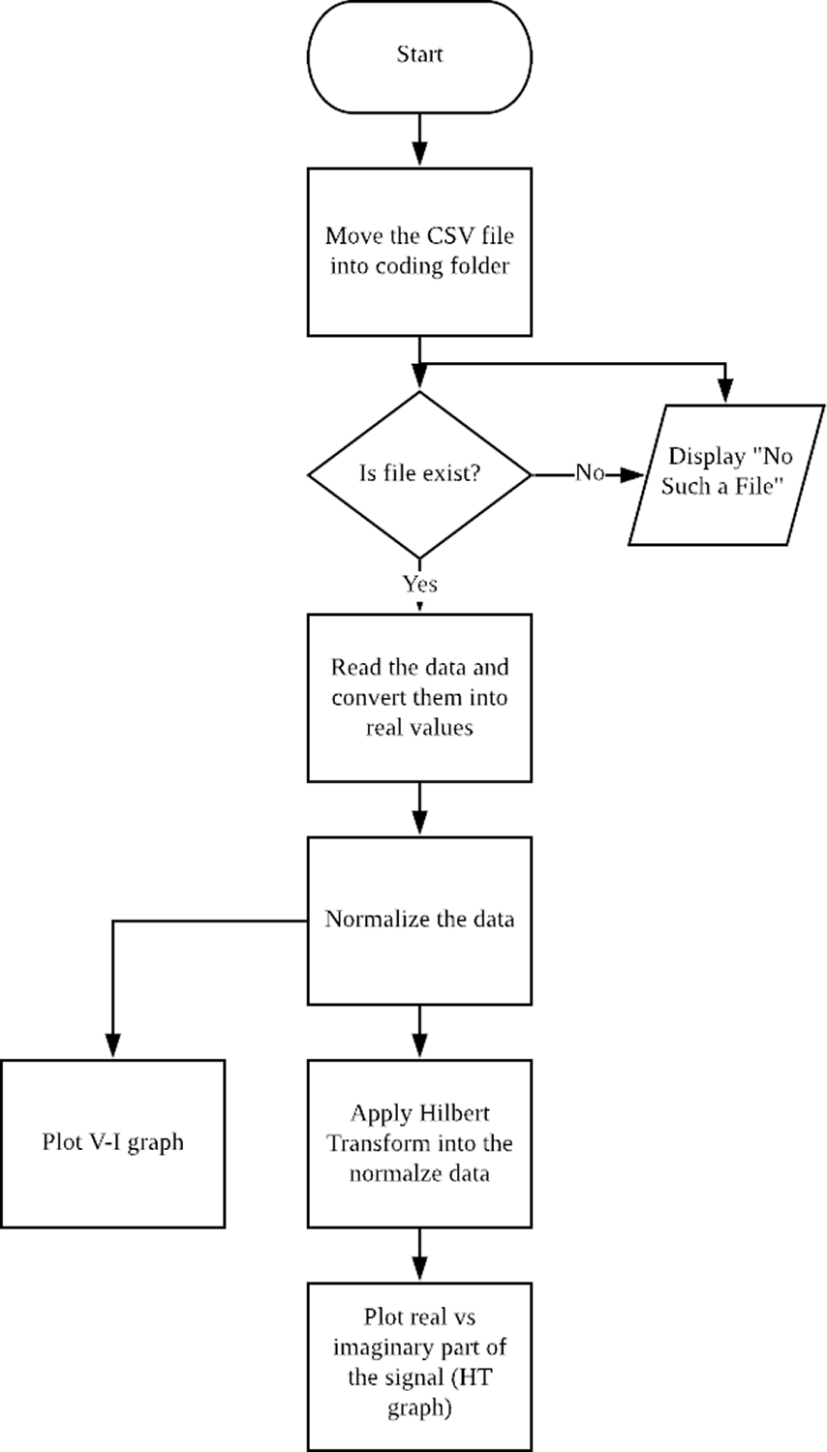
Process of MatLAB/SciLAB coding.

**Figure 4.**
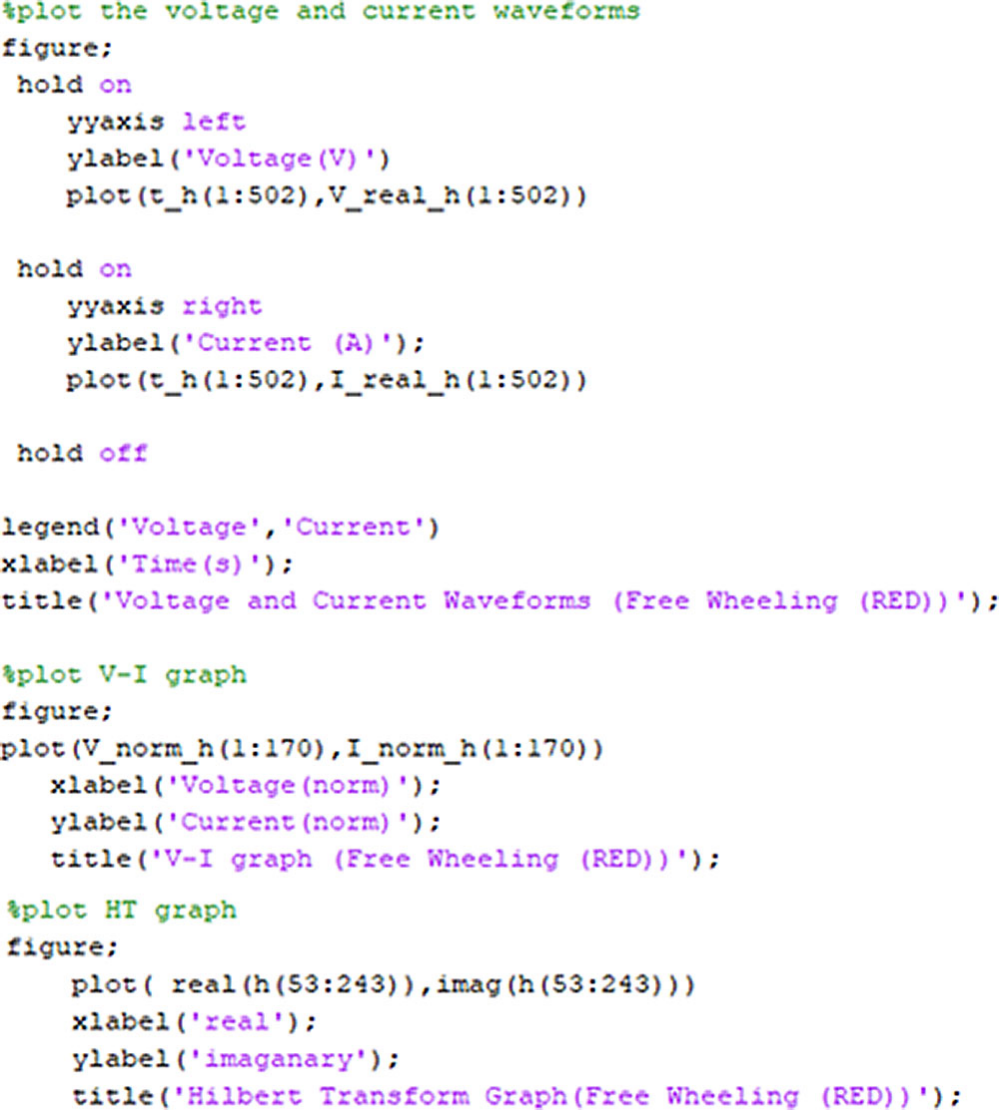
MatLAB/SciLAB part of code for V-I and HT plot.

The potential or voltage sensor (VT) and current sensor (CT) data was captured and stored inside the micro SD card in comma-separated value “.csv” files. The whole process is shown in Figure 3 block diagram. The MatLAB version 2019b or SicLAB version 6.0.2 software was used to process the data. The analysis process resulted in two different graphs: HT plot and V-I trajectory plot. Figure 4 shows a portion of the MatLAB/SicLAB code used to plot the two different graphs. The first part of the code is used to plot VI instantaneous curve using the raw VT and CT sensor data of the red phase. The second part of the code used the raw VT and CT sensor data of the red phase normalized to 0 to 1 of amplitude and plotted them as VI Lissajous trajectory. The final part of the code used only the raw CT sensor data of the red phase that is normalized and creates two sets of data, real and imaginary. Both of them are plotted into a Lissajous plot that we refer to as HT of I only plot.

### Phase three: inference, learning and classification of machine condition

For manual user interface detection of motor faults by employees (technicians) viewing our HT plot installed in a computer, this would only involve phases one and two as described above. This process involves the comparison of pre-determined plots of faults of the motor charts, and technicians could compare them with current real-time HT plots.

An automated artificial intelligence (AI) fault detection involves our method’s phase three, where we have used pre-determined charts or plots of faulty and normal running (healthy) motors to classify types of fault automatically without the involvement of humans. ^4^^,^^5^ The Jupyter Notebook (IPython 7.11.1) was used here to train and test the data for further processes. The system also needs the machine learning libraries from scikit-learn version 0.22 with all the algorithms available there. Next, the directories of testing and training data needed to be set up. This involves 10 samples of HT of current plot for each data sample of the motor faults. The 10 samples taken should have minimum deviation or variations between them, and this is repeated for all the five types of faults detected in this paper. It also requires 10 sample of HT of current plot data samples of normal running (healthy) motor for comparison and analysis. Next, disaggregation algorithms were applied to the datasets and boundaries were drawn to classify that the motor was either in a normal running (healthy) or faulty condition. The system would then determine to which dataset the boundaries belong to according to the similarities, by comparing the figures inside training dataset. Increasing the amount of data trained would significantly increase the system accuracy. There are three algorithms ^21^ used to disaggregate the data sample: vector machine, ^22^ naïve Bayes ^23^ and k-nearest neighbours. ^24^ This would print the predictions based on their respective algorithms. Lastly, the result of machine learning was produced based on the training of the 10 samples. In order to increase its accuracy, as much data needed to be collected as possible.

## Results

### Healthy conditions of pump system (mode 1)

**Figure 5.**
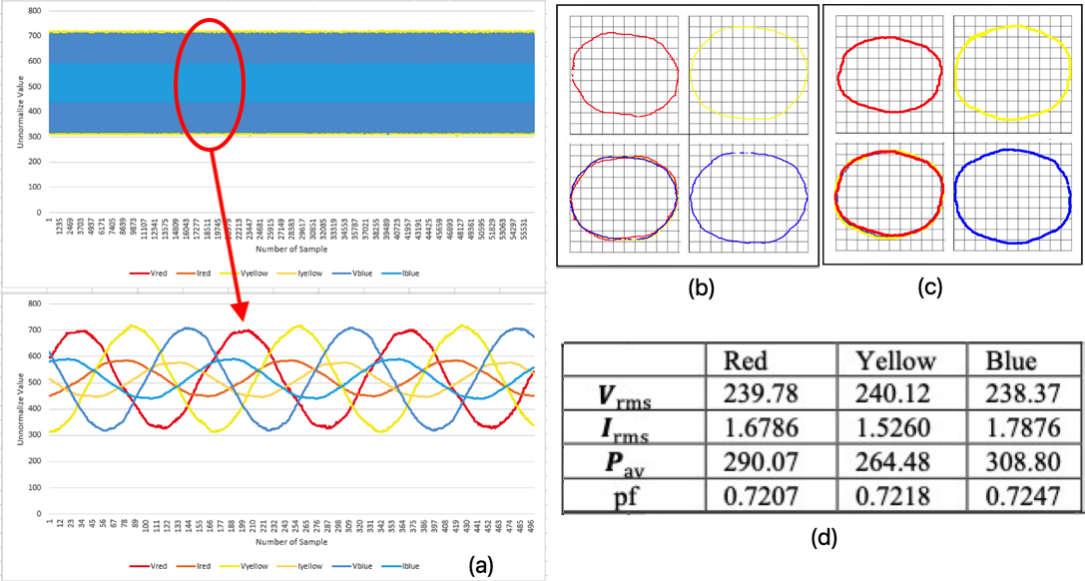
MODE 1 (a) VI instantaneous (b) VI Lissajous trajectory (c) HT of I only (d) Sets calculated via MATLAB.

Figure 5a shows the three-phase instantaneous current and voltage raw data samples taken for a normal running at healthy conditions of a three-phase motor. Approximately 56000 samples of VT and CT were taken in 6.5 seconds. The V-I trajectory and HT graph were put into one plane according to its phase. Based on the observations, there was a small gap in between each of the phases, where it determined the differences in voltage and current in the system were quite small when the graphs were overlaid on each other. The shapes from each phase merely look alike and appear as round or oval shapes. Hence, these observations indicated the system was in a healthy condition. Figure 5c, which used only our HT current signatures curve method, shows the same result observed in Figure 5b using Lissajous voltage and current signatures curve. ^25^

### Open line fault trip at the second phase while running (mode 2)

**Figure 6.**
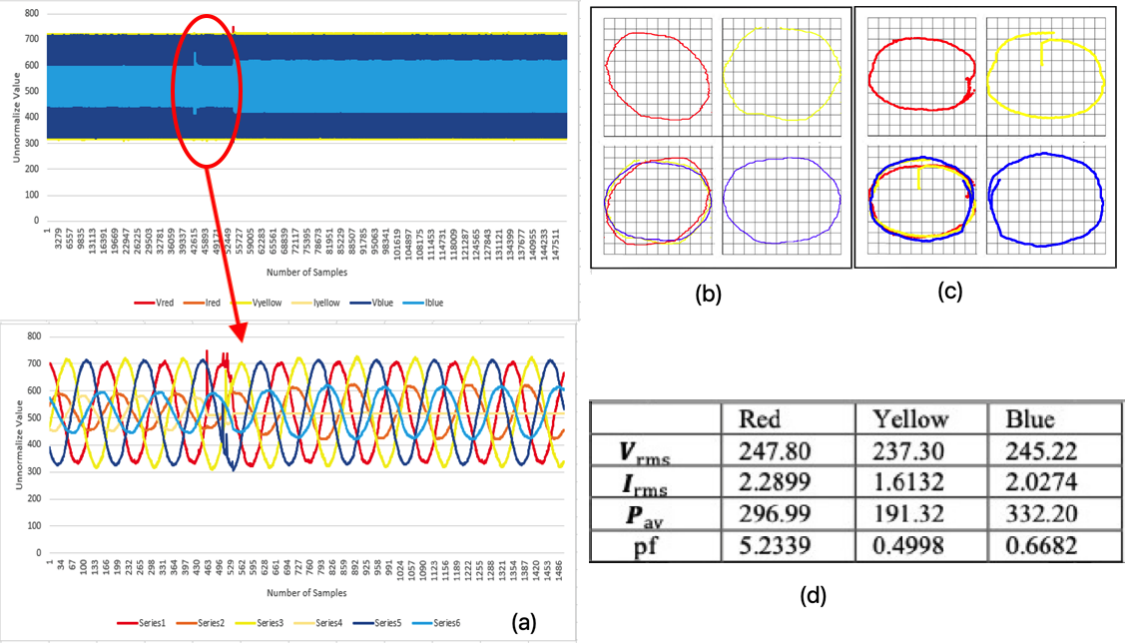
MODE 2 (a) VI instantaneous (b) VI Lissajous trajectory (c) HT of I only (d) Sets calculated via MATLAB.

The three-phase 1-2-3 is represented in this paper as red-yellow-blue colours. Figure 5a shows the three-phase instantaneous current and voltage raw data sample that were taken, approximately 150000 samples of VT and CT in 6.5 seconds for mode 2 faulty motor. The fault was performed by allowing the motor to run in normal condition for approximately 5.3 seconds, before the second sequence phase (yellow phase) was pulled out of the motor terminal. Figure 6a shows that some noise occurred after the yellow phase was pulled out. The yellow phase current was completely out and became zero, which affected the other two remaining phases.

The V-I trajectory and HT graphs of Figures 6b and c, respectively, clearly show some distortion and difference in their shapes when compared with the normal running (healthy) condition (mode 1) in Figure 5. The bottom left quadrant of Figures 6b and c shows the combined phase graph that represents the different behavior of each graph right after the yellow phase was pulled out. Then, it was observed that there were gaps in between each phase when they were put in one plane. The gaps proved the difference in voltage and current in the system that appeared when the graphs were overlapped to each other. This observation indicated the system was in a faulty condition since there were gaps and differences when compared with the healthy condition. Figure 6d shows the value calculated from MATLAB obtained from the raw data. The value of maximum voltage and current were increased slightly due to some noise occurring after the yellow phase was pulled out.

### The motor started without second phase (mode 3)

**Figure 7.**
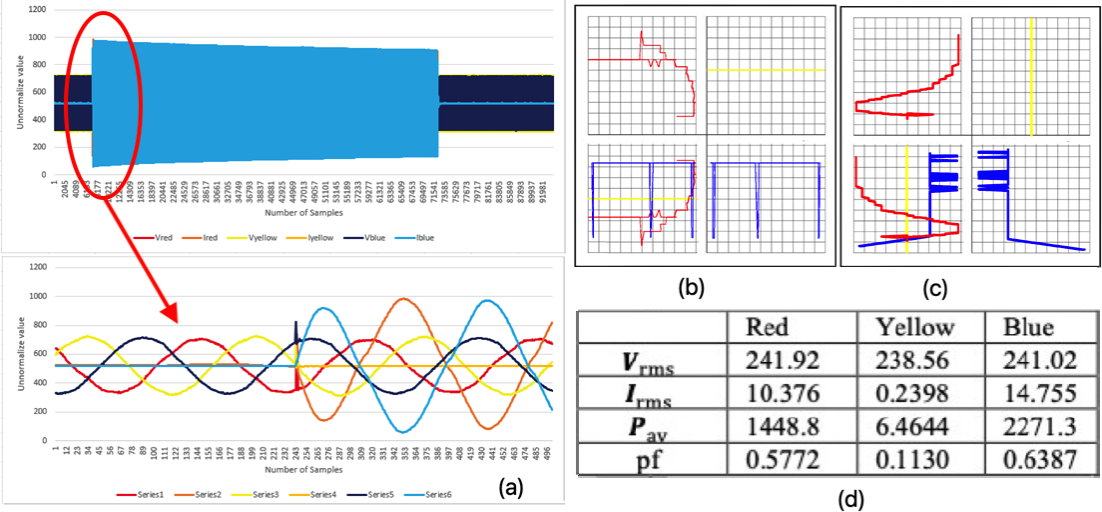
MODE 3 (a) VI instantaneous (b) VI Lissajous trajectory (c) HT of I only (d) Sets calculated via MATLAB.

The phases extracted from the graphs, as are shown in Figure 7a, show the condition of each phase voltage and current taken, approximately 95000 samples in 6.5 seconds for mode 3 faulty motor. The currents for all the phases were almost zero before the motor starts where else there will be voltage across the motor measured even though motor is in halt position. Then the second sequence (yellow phase) was pulled out just before starting the motor as shown in the Figure 7a. The current was increased instantly during this time, while the voltage waveform became a bit noisy in a fraction of a second (transient). There were large differences between each of the phases in Figures 7b and c where these determined the difference of voltage and current in the system after the graph overlapped with each other. The shapes produced from the HT and V-I trajectory were very different from the oval shape displayed by the normal running condition (mode 1), due to the transient effect after the yellow phase was pulled out. Therefore, this indicated that the system is in fault condition. The numerical data calculated from MatLAB in Figure 7d showed a different value pattern with the healthy condition. The value of the maximum current rose to extremely high levels compared to the normal current condition (mode 1).

### Motor protection trips due to high current (mode 4)

**Figure 8.**
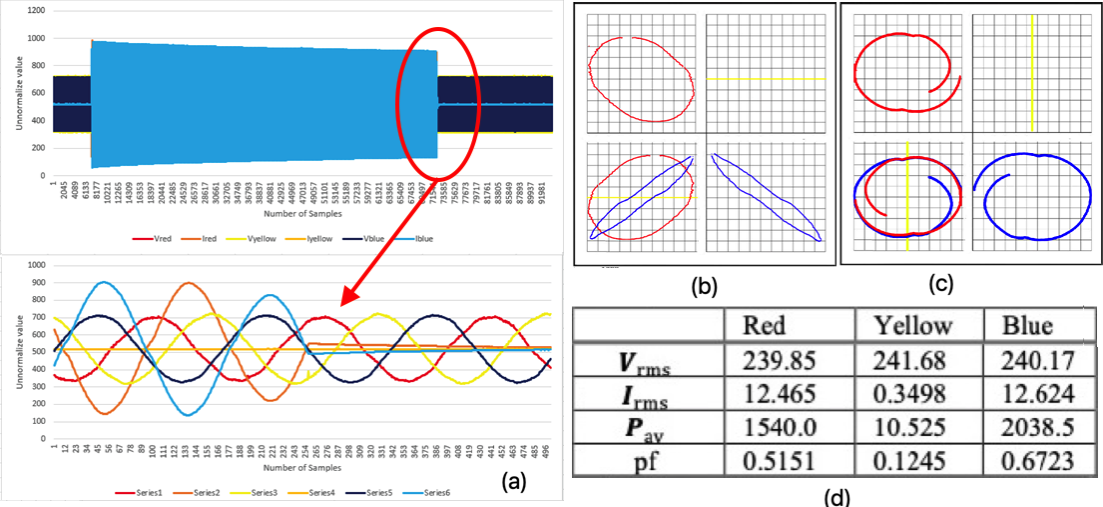
MODE 4 (a) VI instantaneous (b) VI Lissajous trajectory (c) HT of I only (d) Sets calculated via MATLAB.

Figure 8a shows the three-phase instantaneous current and voltage raw data samples that were taken, approximately 95000 samples of VT and CT in 6.5 seconds for mode 4 faulty motor. Initially the current for all phases was increased to almost double the normal current, as shown in Figure 8a. Once the fault mode 3 was present, the current suddenly increased, for the other two phases but the rotor of the motor did not turn off as one of the phases was already being pulled out. Figure 8a shows the three-phase instantaneous current and voltage raw data sample that were taken, approximately 56000 samples of VT and CT in 6.5 seconds for mode 4 faulty motor. During these few seconds, the motor became stressed due to the high current and the protection immediately cut off the power supply to avoid damage to the equipment. The current of all phases fell to zero once the protection tripped. Based on the graph observation in one plane as shown in Figures 8b and c, there were peculiar shapes in every phase except for the red phase, and in between each phase where it determined the difference in voltage and current in the system after the graphs were overlapped with each other. The shapes from each phase showed an identical pattern and were almost the same as the distorted shape, except for yellow phase which showed only straight horizontal lines due to there being no current. This observation indicated the system was in a faulty condition. Figure 8d shows the value of the maximum current rose to extremely high levels compared to the normal current (mode 1) condition, where the high current in red and blue phases caused the trip in the protection.

### Healthy condition of induction motor with brake pulley (mode 5)

**Figure 9.**
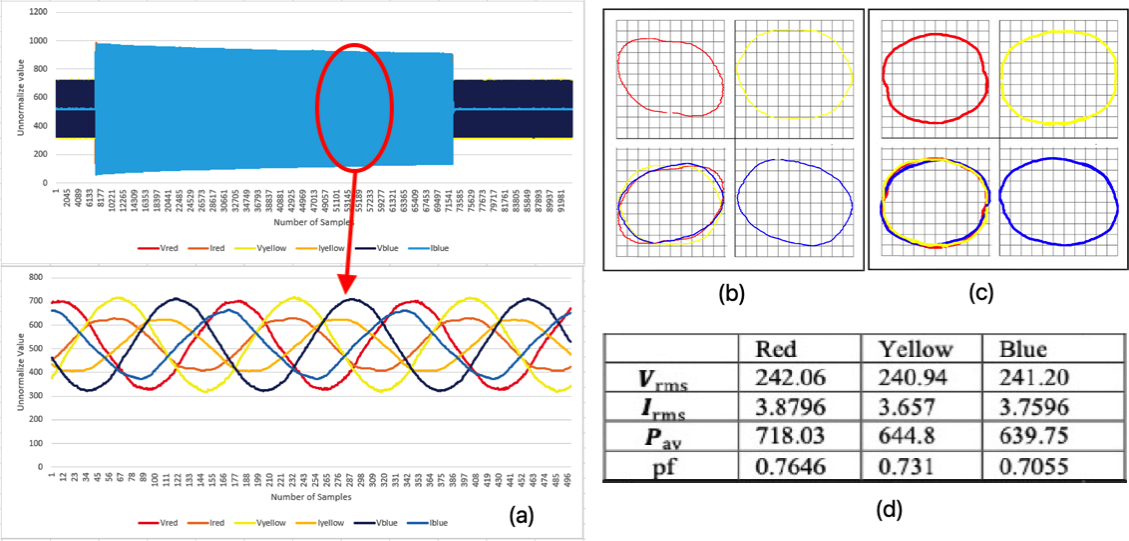
MODE 5 (a) VI instantaneous (b) VI Lissajous trajectory (c) HT of I only (d) Sets calculated via MATLAB

Figure 9a plot shows approximately 46000 samples taken in 10.8 seconds with unnormalized values of three phase voltage and current respectively. It shows a normal condition with normal running (mode 1) condition of the motor. The shape of the V-I trajectory and HT of Figures 9b and c are round; these were the normal condition with no distortion on the shapes. The values of the voltage and current from each phase were only slightly different to each other, as shown in Figure 9d.

### Voltage varied in tighten (loaded) condition (mode 6)

**Figure 10.**
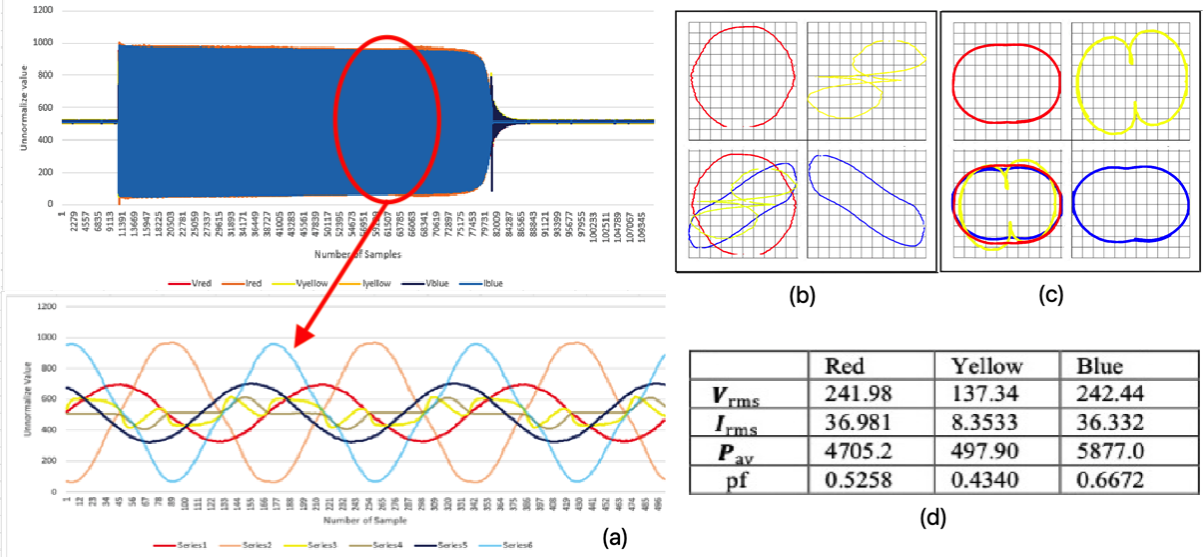
MODE 6 (a) VI instantaneous (b) VI Lissajous trajectory (c) HT of I only (d) Sets calculated via MATLAB.

For mode 6, the voltage was varied by using the auto-transformer (Siemens model IBC 3P-15kW) where the second phase sequence (yellow phase) voltage was varied in a loaded condition. The voltage was varied from the standard voltage to a slightly lower voltage and then increased to be slightly higher. The graph in Figure 10a shows approximately 115000 samples taken in 10.8 seconds, where there are some variations of current and voltage in the yellow phase, as the voltage was in an unbalanced system. Based on this observation, it appears there was some unusual noise occurring while the voltage was explicitly varied on the yellow phase. However, the other two phases were not affected by this yellow phase. After data was analyzed by MatLAB, the graphs were put in one plane according to its phase as shown in Figures 10b and c. It was observed that there were different kinds of shapes that appeared in between each of the phases, where it determined the differences in voltage and current in the system for each phase after the graphs were overlapped with each other. The red phase showed a closed to round shape while the other two phases showed superimposed with two oblong oval shape. This indicated the faulty condition of the system. Based on Figure 10d, the current for red and blue phases had increased to almost 10 times their normal value as the yellow phase voltage lowered with auto-transformer.

## Conclusions

This project involves hardware, programming and data analysis software. We have successfully conducted and completed the experiment for two three-phase induction motors with four fault simulations in a laboratory environment. There are many types of faults that can be monitored and identified but we have only chosen these four types as they could be simulated without damaging the experiment hardware sets in the campus laboratory. In this project, it was found that our HT instantaneous current signature fault detection method is a better choice in a disaggregation approach, as it was able to show obvious differences in healthy and faulty curves. The better diversity in the visualization of fault curves helps to increase the accuracy of the interpretation of AI software, and will help a maintenance crew to pinpoint or identify the fault in real time via LCD monitors as each fault will give a specified fault curve signature. From the experiment, we conclude that our system is also able to identify pre-faults. This pre-fault can be detected when a slight defection starts to occur towards a pre-determined known fault curve in our database. Detection of pre-faults would help the maintenance period by pre-stocking parts needed with scheduled downtime; otherwise, unscheduled downtime will occur with will incur losses in production profit for industries that rely on this main horsepower. Another big advantage in our system is the hardware cost for the sensor is reduced by 50%, as only three current transformers are needed, while other systems in the market need another three potential transformer sensors. For future work we would recommend testing our system design in an actual production factory environment for three-phase induction motors, synchronous motors and other types of motors.

## Data availability

### Underlying data

Zenodo: drshashikumar/rawdata: Raw data Excel. https://doi.org/10.5281/zenodo.5229187. ^25^

This project contains the following underlying data:

- Mode1.csv: Healthy motor Mode 1, raw data excel file three-phase for voltage and current
- Mode2.csv: Motor fault Mode 2, raw data file
- Mode3.csv: Motor fault Modes 3, raw data file
- Mode4.csv: Motor fault Modes 4, raw data file
- Mode5.csv: Motor fault Mode 5, raw data file
- Mode6.csv: Motor fault Mode 6, raw data file
- readme.txt: data key

Data are available under the terms of the Creative Commons Zero “No rights reserved” data waiver (CC0 1.0 Public domain dedication).
