# Supplementary material for: Detecting industrial motor faults with current signatures
Source: F1000Res. 2021 Sep 8;10:903. [Version 1] doi: 10.12688/f1000research.54266.1 (PMC9634139; doi:10.12688/f1000research.54266.1)
Supplement: Peer review history

# Peer review: Reviewer response for version 1

**DOI:** 10.5256/f1000research.57736.r151359

**Published:** 2022-11-03

**Authors:** Chandima Gomes

**Affiliations:** 1 University of the Witwatersrand, Johannesburg, South Africa

This paper presents a relatively novel method of detecting 3-P industrial motor faults, that requires only Hilbert Transform (HT) instantaneous current signature curve as the input parameter. The outcomes have been compared with the results obtained from other methods. The paper is technically sound. However, there are redundant sentences such as the very first sentence of the abstract. It is recommended to remove such. I would also like to recommend the author pay attention to the way of presenting the information. Although the English grammar is acceptable, the clarity of the presentation of outcomes could vastly be improved.

Is the work clearly and accurately presented and does it cite the current literature?

Yes

If applicable, is the statistical analysis and its interpretation appropriate?

Not applicable

Are all the source data underlying the results available to ensure full reproducibility?

Yes

Is the study design appropriate and is the work technically sound?

Yes

Are the conclusions drawn adequately supported by the results?

Yes

Are sufficient details of methods and analysis provided to allow replication by others?

Yes

Reviewer Expertise:

Electrical and high voltage engineering

I confirm that I have read this submission and believe that I have an appropriate level of expertise to confirm that it is of an acceptable scientific standard.

# Peer review: Reviewer response for version 1

**DOI:** 10.5256/f1000research.57736.r93826

**Published:** 2021-09-28

**Authors:** Jeyraj Selvaraj

**Affiliations:** 1 UM Power Energy Dedicated Advanced Centre (UMPEDAC) Wisma R & D, University of Malaya, Kuala Lumpur, Malaysia

This work involves monitoring faults in three-phase industrial motors Hilbert Transform instantaneous current signature curve. The system detects fault signature accurately at any voltage and current levels. The authors may address these comments in order to improve the manuscript:

1. Where would be the possible implementation of this method?
2. How robust is the system compared to conventional systems?
3. Is there any increase or decrease in the efficiency of the motor by adopting this method?
4. Can the method be used in other motors besides induction motors?

Is the work clearly and accurately presented and does it cite the current literature?

Yes

If applicable, is the statistical analysis and its interpretation appropriate?

Yes

Are all the source data underlying the results available to ensure full reproducibility?

Yes

Is the study design appropriate and is the work technically sound?

Yes

Are the conclusions drawn adequately supported by the results?

Yes

Are sufficient details of methods and analysis provided to allow replication by others?

Yes

Reviewer Expertise:

Power Electronics and Drives

I confirm that I have read this submission and believe that I have an appropriate level of expertise to confirm that it is of an acceptable scientific standard.
